# Toward Characterizing Lymphatic Vasculature in the Mammary Gland During Normal Development and Tumor-Associated Remodeling

**DOI:** 10.1007/s10911-023-09554-w

**Published:** 2024-01-13

**Authors:** Petra Dahms, Traci R Lyons

**Affiliations:** 1 Division of Medical Oncology Senior Scientist, Young Women’s Breast Cancer Translational Program, University of Colorado Cancer Center, 12801 E 17th Ave, RC1 South, Mailstop 8117, 80045 Aurora, CO USA; 2 Division of Medical Oncology, Anschutz Medical Center, University of Colorado, Aurora, CO USA; 3 Anschutz Medical Campus Graduate Program in Cancer Biology, University of Colorado, Aurora, USA

## Abstract

Lymphatic vasculature has been shown to promote metastatic spread of breast cancer. Lymphatic vasculature, which is made up of larger collecting vessels and smaller capillaries, has specialized cell junctions that facilitate cell intravasation. Normally, these junctions are designed to collect immune cells and other cellular components for immune surveillance by lymph nodes, but they are also utilized by cancer cells to facilitate metastasis. Although lymphatic development overall in the body has been well-characterized, there has been little focus on how the lymphatic network changes in the mammary gland during stages of remodeling such as pregnancy, lactation, and postpartum involution. In this review, we aim to define the currently known lymphangiogenic factors and lymphatic remodeling events during mammary gland morphogenesis. Furthermore, we juxtapose mammary gland pubertal development and postpartum involution to show similarities of pro-lymphangiogenic signaling as well as other molecular signals for epithelial cell survival that are critical in these morphogenic stages. The similar mechanisms include involvement of M2-polarized macrophages that contribute to matrix remodeling and vasculogenesis; signal transducer and activator of transcription (STAT) survival and proliferation signaling; and cyclooxygenase 2 (COX2)/Prostaglandin E2 (PGE2) signaling to promote ductal and lymphatic expansion. Investigation and characterization of lymphangiogenesis in the normal mammary gland can provide insight to targetable mechanisms for lymphangiogenesis and lymphatic spread of tumor cells in breast cancer.

## Introduction to Lymphatic Vasculature Structure and Development

The lymphatic vascular system functions to maintain homeostatic fluid and immune cell maintenance and surveillance in the body. Lymph fluid contains tissue-derived cell debris, immune cells, and/or other cells or cell components. Lymph is trafficked by lymphatic vessels for removal and monitoring of tissue homeostasis by lymph nodes. Lymphatic vasculature is organized based on vessel size and function. Capillary lymphatic vessels pick up lymph fluid containing cells and debris, transporting these tissue components to the larger collecting lymphatics. Lymphatic capillaries are specifically designed to carry out this function via their specialized endothelial cell junctions, termed “button-like” junctions. Button-like junctions contain gaps between the lymphatic endothelial cells (LECs) that allow for movement of lymph through the vessel walls [1, 2]. These gaps are also large enough for cells, such as immune cells, to pass through with the lymph. These specialized endothelial cell junctions contain adherence junctions and tight junctions. Adherence junctions are characterized by their expression of Vascular Endothelial (VE)-Cadherin which binds through β-catenin to the cytoskeleton [3]. Tight junctions are characterized by their expression of Zonula Occludens 1 (ZO1) expression, which regulates VE-Cadherin junctions [3]. The gaps between individual cell junctions get closer together as the lymphatic vessels increase in size, with capillary lymphatics transforming into collecting lymphatics with tight “zipper-like” junctions [1]; collecting lymphatics are also supported by a lining of smooth muscle cells that contract to help pump lymph toward the lymph node and valves that ensure unidirectional lymph flow. These collecting lymphatics are connected to a series of lymph nodes; Lymph nodes contain immune cells, such as macrophages, dendritic cells, T cells and B cells, that survey lymph fluid for particles and/or pathogens to be eliminated [4]. Once filtered by the lymph nodes, the lymph returns to the blood vascular system by routing through the right and left thoracic ducts, which connect to the subclavian vein [4, 5]. While this specialized system allows for immune cell uptake and transportation, this system also allows lymphatic vessels to transport tumor cells to other parts of the body [6].

Initial lymphangiogenesis occurs during embryogenesis, with undifferentiated endothelial cells arising from the mesoderm, and a lymphatic sac budding from the cardinal vein to form the initial lymphatic vessels at embryonic day 9.5 in mice [2, 7, 8]. Transcription factor prospero homeobox protein 1 (PROX1) expression is necessary for LEC differentiation, as its expression determines lymphatic fate of endothelial cells during early differentiation [9, 10]. Furthermore, PROX1 is necessary and sufficient for LEC fate as downregulation promotes blood endothelial cell (BEC) identity and overexpression is sufficient to drive LEC fate and expression profiles [11–14]. After LEC differentiation, vessel formation begins with the establishment of pro-lymphangiogenic molecular signaling. The main pro-lymphangiogenic molecules identified to date are members of the vascular endothelial growth factor (VEGF) and receptor (VEGFR) families. VEGF signals through VEGFR to activate canonical pathways to promote cell survival, proliferation, invasiveness, and permeability–like what is known from angiogenesis. VEGFC and VEGFD are the two major family members characterized as pro-lymphangiogenic. VEGFC/D molecules are expressed by epithelial cells as well as stromal cells, including blood vascular and lymphatic endothelial cells, macrophages, and fibroblasts, while their receptors, VEGFR2/R3, are found almost exclusively on blood vascular and lymphatic endothelial cells [15]. VEGFC/D signaling through VEGFR2/R3, which are tyrosine kinase receptors, results in activation of protein kinase B (PKB), or AKT, and downstream extracellular signal-regulated kinase 1/2 (ERK1/2) pathways [16]. Activation of AKT/ERK pathways leads to LEC migration and survival [16, 17]. Insight into the relative roles of VEGFC/D during initial development has been gained through studies of transgenic mice where *Vegfc-/-* mice failed to form lymphatic vessels [18] while *Vegfd-/-* only exhibited minor defects [19]. These studies suggest that VEGFC protein is required for initial development of lymphatic vasculature in the embryo, while VEGFD is likely more important for upkeep of lymphatic vasculature in the adult. Additionally, lymphatic cell-specific Cre-knockout of *Vegfr2* gene in mice showed reduced lymphatic network formation but did not affect lymphatic vessel maturation or function [20]. Finally, VEGFR2 does not induce lymphatic vessel sprouting in adult mice, while VEGFR3 activation through VEGFC/D does [21]. VEGFR3 also maintains a positive feedback loop for *Prox1* gene expression to help maintain LEC progenitors during embryogenesis in mice [22]. In summary, VEGFR3 appears to be the dominant receptor for pro-lymphangiogenic signaling during embryogenesis and in adult mice.

Other implicated factors in lymphangiogenesis include neuropilin molecules, NRP1 and NRP2–transmembrane glycoproteins that associate with VEGF and VEGFR family members and can activate the downstream signaling cascades to modulate LEC migration. While NRP2 binds to VEGFR3 [23] and interacts with VEGFC and VEGFD in LECs to promote lymphangiogenesis [24], NRP1 also associates with receptors PlexinA1 and VEGF to promote lymphatic function and valve formation [25, 26]. Valve formation and smooth muscle cell association in collecting lymphatics is also promoted by transcription factor forkhead box C2 (FOXC2) [27] and growth factor ligand Angiopoetin 2 (ANG2) signaling through its tyrosine kinase receptor (TIE2) [28]. Furthermore, FOXC2 regulates cytoskeleton organization to stabilize LEC junctions and vessel structure in response to shear stress [29]. The ANG2/TIE2 receptor ligand interaction also serves to stabilize the zipper-like LEC junctions observed in collecting lymphatics [28]. An additional well-known lymphatic vessel-associated protein is podoplanin (PDPN), which marks LECs in the lymph node and in the collecting lymphatics. PDPN is necessary for maintenance of lymphatic vascular structure [30] and further differentiates LECs from BECs during embryonic development. *Pdpn* gene expression has also been implicated during development of various organs, including the heart and lungs, for cell proliferation and motility [31]. A knockout of PDPN shows deformed lymphatic vessel structure and flow [32]. While FOXC2, ANG2/TIE2, and PDPN mark collecting lymphatics, lymphatic capillaries have other specific markers. For example, chemokine CCL21 allows for recruitment of dendritic cells toward lymphatic capillaries during inflammation [2, 33]. Additionally, a frequently used LEC surface marker is lymphatic vessel endothelial hyaluronan receptor 1 (LYVE1) [2, 34]. LYVE1 is a glycoprotein receptor that binds to hyaluronan and is a proposed marker of neo-lymphatics or lymphatic capillaries. While downstream signals activated by LYVE1 as a hyaluronan receptor on LECs are not well characterized, LYVE1 is a homolog of CD44, which is also hyaluronan receptor known to activate lymphocytes, direct circulation of lymphocytes, and aid in tumorigenesis and lymph node metastasis in breast cancer [35–39]; thus, LYVE1 may serve a similar function as CD44 in LECs. Although the expression of PDPN and LYVE1 are found on lymphatic vessels, lymph nodes, and some blood vessels, we and others have identified PDPN and LYVE1 expression on a population of macrophages that associate with lymphatic vasculature, termed PoEMs, or Podoplanin-expressing macrophages [40]. This topic was covered extensively in a previous review on these specialized macrophages in the mammary gland [41]. PoEMs support lymphangiogenesis by developing into a pseudo-endothelial cell and incorporating into the lymphatic vessel structure in a manner that is dependent on PDPN signaling through C-type lectin-like type II receptor (CLEC2) [42]. In a mouse mammary tumor model, PoEMs localize near tumor-associated lymphatic vasculature and promote matrix remodeling, lymphatic sprouting, and tumor cell metastasis [40]. In addition to mammary remodeling and mammary tumorigenesis, “vascular mimicry” by macrophages has been reported in various neo-lymphangiogenic events in vivo and in vitro, including inflammation [43, 44], wound healing [45], and other tumor-associated lymphangiogenesis [46–48]. While we have primarily focused on identifying PoEMs in the context of breast cancer-associated lymphangiogenesis, further characterization and identification of PoEMs during mammary morphogenic events—such as during establishment of initial lymphatic formation during embryogenesis and as the mammary gland outgrows during pubertal development—is warranted and an ongoing investigation in our lab. It is also important for studies to be undertaken to understand whether PoEMs differentiate from an influx of circulating monocytes and/or from resident mammary macrophages. Finally, in addition to venous-originated lymphangiogenesis, lymphatic vasculature also arises via hemogenic endothelial cells. During this process, endothelial cells spatially cluster together to form spontaneous lymphatic vessels in a process known as lymphvasculogenesis [49]. While lymphvasculogenesis has not been identified in the mammary gland, it may be proposed as an alternative origin of lymphatic vessels that may more easily incorporate other cell types, such as macrophages. This idea will be discussed later in this review.

## Overview of Lymphatics and Mammary Gland Development

The mammary gland is first established during embryogenesis with formation of mammary rudiments from the mesenchyme in the mouse by embryonic day 10–12 [50]. Left-right asymmetry of the mammary line formation leads to independent development of paired mammary glands in the mouse with each gland having differential developmental signaling and expression patterning [51]. In humans, the establishment of the mammary gland bud begins as early as week 5 of gestation, and formation of mammary rudiments continues throughout gestation into early newborn stages and 4 weeks postpartum [52, 53]. However, this initial functional and morphological evolvement of the female breast tissue persists until 2 years of age, after which the glands remain quiescent until puberty which occurs on average from ages 8.5 to 14 years [54]. The mammary rudiments expand during puberty to form a ductal tree, where hormonal signaling—including growth hormone (GH), prolactin (PRL), insulin-like growth factor (IGF) 1, and estrogen-mediated activation of the estrogen receptor (ER)—encourages elongation of the terminal end buds (TEBs) located at the tip of the mammary ducts [50]. The TEBs are surrounded by a layer of cap cells that lead invasion into the mammary fat pad [53, 55]. Bifurcation and side branching of the TEBs occur to create a vast ductal network infiltrating the mammary fat pad [50]. This branching and elongation are regulated by several growth factors, such as amphiregulin (AREG), transforming growth factor beta 1 (TGFβ1), and epidermal growth factor (EGF) [50]. AREG can promote proliferation of LECs in mice [56]; TGFβ1 stabilizes vessel structure in mice [57]; and EGF receptor (EGFR) promotes lymphangiogenesis in normal development and tumor mouse models [56, 58–60]. As elongation of the ducts occurs, highly proliferative ductal epithelial cells trail behind the invading cap cells with the inner epithelial cells forming a cleared lumen. These luminal epithelial cells are encapsulated by cap cells that differentiate into myoepithelial cells [55]; thus, forming the final functional structure of the mammary gland for the future stages of mammary morphogenesis.

The lymphatic network in the mouse mammary gland is first established during embryogenesis and undergoes remodeling along with the mammary gland throughout mammary morphogenic events. Evidence suggests that lymphatic vasculature aids in surveillance of the mammary tissue by immune cells as well as by functioning to clear away cell debris during these developmental stages. In humans, axillary lymph nodes drain 75–80% of lymph from breast tissue, demonstrating the importance of lymphatic development during mammary morphogenesis [61]. During early pregnancy, mouse mammary lymphatic vessel density (LVD) is increased and corresponds to increases in VEGFC and VEGFD expression [62]. The increased VEGFC/D expression then decreases during late pregnancy and lactation [63]. Furthermore, LVD, as measured by LYVE1 + or PROX1 + per mm^3^, was found to decrease during lactation compared to pregnancy [62]. This lack of lymphatic vessel identification may be explained by enlargement of ducts during lactation, making visualization of the vessels difficult. Mouse mammary lymphatic vessels are also found not associated with mammary alveoli during pregnancy and lactation, though lymphatic vessels in proximity to milk-producing alveoli could physically limit and block the milk supply [63]. Furthermore, enlarged intramammary and axillary lymph nodes during lactation in human patients can be detected via mammography [64], indicating immune surveillance of the mammary gland. This active immune surveillance through lymphatic vasculature is additionally supported by identification of a specialized subset of macrophages present in human milk during lactation that show a strong immune response in mice [65]. After lactation is complete and involution begins, there is an increase in apoptotic cell debris from the remodeling events [66], which is likely to be cleared from the mammary gland through the lymphatic vasculature. During the first, reversible phase of involution, VEGFC remains low, along with LVD [62, 67]. During the second, irreversible phase, VEGFC is increased two-fold [62]. This increase in VEGFC coincides with an increase in VEGFR2/3 [62], leading to a remodeling of the lymphatic network during involution. A more in-depth review of postpartum lymphatics has been published [62]. Importantly, the new lymphatic structures that develop during involution have been postulated to persist in women up to 10 years post-partum and may aid in progression of postpartum breast cancers (PPBCs) [62]. VEGFC has also been shown to recruit tumor-associated macrophages (TAMs) into the mammary gland in mice [68]. Therefore, VEGFC could play a similar role during mammary morphogenic events by helping to recruit macrophages for remodeling and debris clearance during puberty, involution, and tumorigenesis. For example, blood serum from human lipedema patients had increased systemic VEGFC, which may have contributed to an increase in macrophage infiltration, yet there was no discernable change in LVD found in corresponding patient lipedema tissues [69]. These macrophages were identified as a subpopulation with overexpression of CD163, a scavenger receptor, which, when expressed on macrophages, aids in inflammation resolution [70]. CD163 expression is also associated with TAMs [71].

Lymphangiogenesis has yet to be fully characterized during pubertal development, which is when the mammary gland matures to be fully formed and posed to respond to the hormones of pregnancy. One study, by Betterman et al., investigated lymphangiogenesis in the post-embryonic developing mammary gland in mice [63]. Lymphatic vessels were found alongside and spiraled around elongating mammary ducts, like blood vessels, demonstrating that signaling from the mammary ductal cells may regulate lymphangiogenesis during this expansion event. LVD is also increased in mammary glands of MMTV-PyMT mice—a well-established mouse model characterized by increased epithelial cell proliferation that results in spontaneous tumors. This increase in LVD implies an increase in lymphangiogenic growth and/or patterning factors coinciding with the epithelial cell proliferation and expansion like what occurs during puberty [72]. Moreover, a mouse model with a reduced mammary ductal tree expansion (MMTV-specific Cre-inducible *Gata3* KO) showed reduced LVD, further supporting a correlation between ductal elongation and lymphangiogenesis during pubertal development [63]. Investigation into signaling responsible for this correlation showed increased expression of pro-lymphangiogenic growth factors (VEGFC/D, PDGFA, PDGFB, FGF1, HGF) in myoepithelial cells compared to luminal epithelial and hematopoietic cells. Following pubertal development, VEGFC, and not VEGFD, is the primary pro-lymphangiogenic stimulus in the formation of the pregnancy-associated mammary gland [63]. In a *Vegfd* knockout mouse model, there was no change to ductal branching-associated lymphangiogenesis in the virgin and pregnancy-associated mammary gland, which is similar to what was seen during initial lymphangiogenesis in the *Vegfd-/-* embryo where there were no major changes in lymphatic vessel formation [19]. Overall, it is thought that the myoepithelium of the mammary ducts promotes lymphangiogenesis during post-embryonic development in the mammary gland and this is primarily driven by VEGFC.

Since lymphangiogenesis presumably correlates with proliferation of the myoepithelium, the left-right asymmetry established in the ductal tree formation during embryogenesis [51] could also be important for lymphangiogenesis. While left-right asymmetry of the mammary gland has been primarily investigated in mice and humans, it is thought to occur in most mammals due to bilateral development of mammary gland pairs [51]. This left-right asymmetry of the mammary ductal network leads to laterally-unique gene expression profiles between mammary glands, and can lead to differential oncogene activity and disease progression in right versus left mouse mammary tumors [73]. Furthermore, breast left-right asymmetry in breast volume measured via mammogram is a predicting factor for increased risk of breast cancer in human patients [74]. Therefore, the left-right asymmetry in ductal outgrowth during embryogenesis and pubertal development could establish left-right asymmetry for the lymphatic network in the mammary gland, and go on to similarly establish a basis for asymmetric lymphangiogenesis during tumorigenesis. However, left-right asymmetry remains to be investigated in mammary lymphangiogenesis. For example, it would be of interest to investigate whether a right mammary gland has increased VEGF expression during mammary morphogenesis compared to the left, and if this persists in lateral tumorigenesis and tumor-associated VEGF expression in mice. Additionally, we and others have shown that LVD is increased in breast tissues from recently pregnant women [75] and breast cancers from recently pregnant women, or postpartum breast cancers (PPBCs), have increased tumor associated LVD and increased risk for metastasis [67]. Therefore, since LVD correlates with mammary ductal outgrowth [63], similar asymmetrical lymphangiogenesis during postpartum involution could be an important factor in this increased risk.

**Fig. 1:**
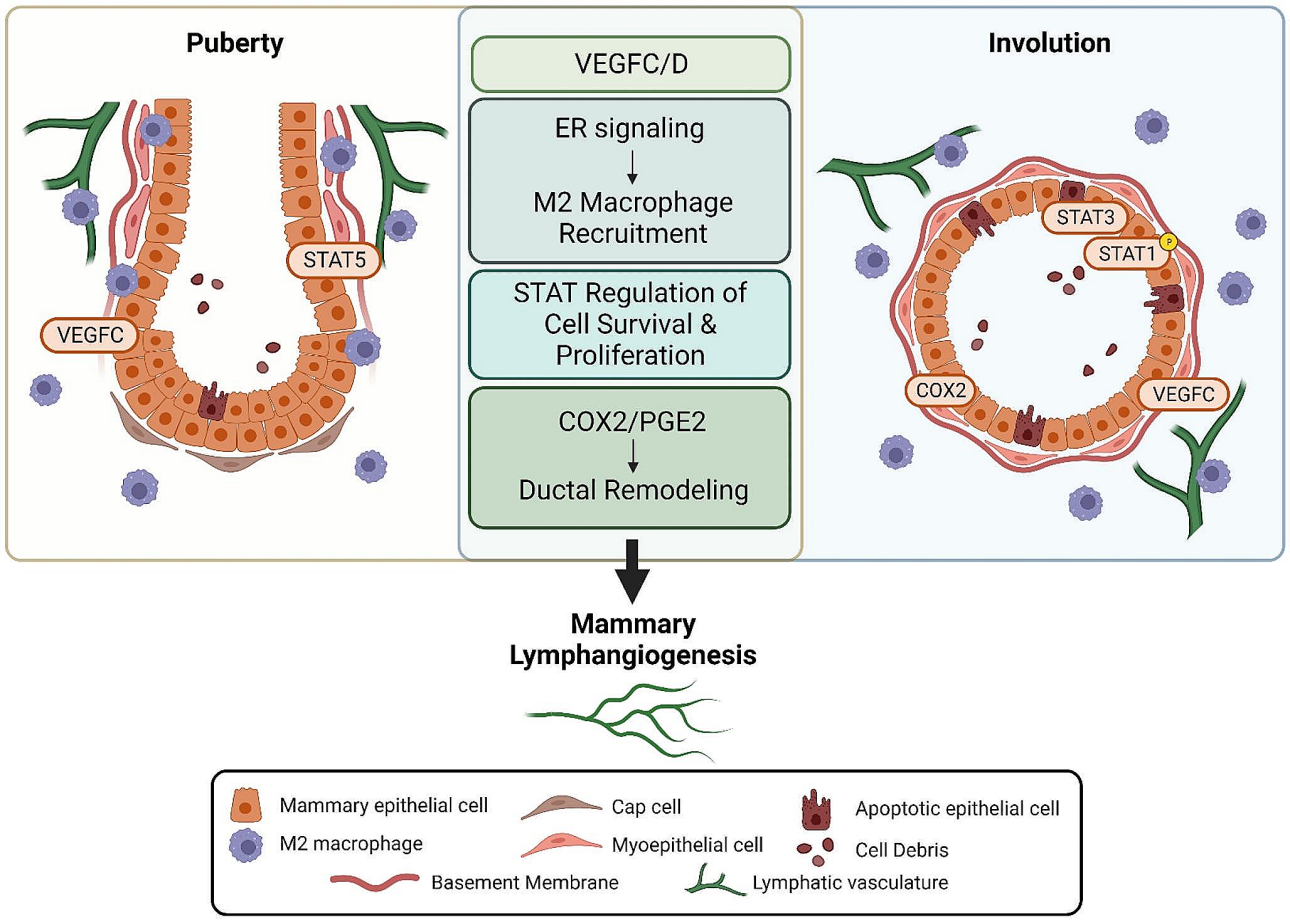
Model demonstrating overlapping signaling mechanisms to promote lymphangiogenesis during mammary gland pubertal development and postpartum involution. Mammary gland pubertal development (left, representative terminal end bud) and postpartum involution (right, representative mammary duct) have increased expression of lymphangiogenic factors VEGFC and VEGFD, with increased dependence on VEGFC. Both morphological events are regulated by estrogen receptor (ER) signaling which has been shown to recruit M2-polarized macrophages; M2-like macrophages have been found to make up the majority of the macrophage population during both events. STAT signaling – STAT5 during puberty and STAT3 and phospho-STAT1 during involution – have been shown to promote epithelial cell survival and proliferation. Finally, the COX2/PGE2 signaling cascade has been shown to regulate ductal elongation and matrix restructuring, which has been shown to regulate lymphatic vessel organization. Overall, these factors are all suggested to promote lymphangiogenesis during these developmental stages. This figure also demonstrates cellular similarities occurring during remodeling, including apoptotic epithelial cells, and epithelial cell signaling occurring during each stage. Figure was created using BioRender.com.

**In summary**, although the role for lymphatic vasculature in the developing mammary gland during puberty is less well-studied, investigating the similarities between the remodeling events that occur during puberty and involution, including epithelial cell survival and stromal cell matrix remodeling, may lead to additional insights. By comparing these remodeling events (Fig. 1), we may further understand lymphangiogenic signaling that normally occurs in the mammary gland, which may lead to a deeper understanding of tumor-associated lymphangiogenesis. In the next few sections we will summarize cells and signals that orchestrate these remodeling events.

## Macrophages

Macrophages are known to play a variety of roles in a variety of tissues and are typically identified on a “polarized” spectrum of function depending on expressed and secreted markers [76–78]. M1-polarized macrophages (CD38 + iNOS + TNFα + IL-1 + IL-6+) are known to play a pro-immunity role during immune responses such as pathogen recognition and clearance; M2-polarized macrophages (CD206 + ARG1 + IL-10 + IL-4+) are known to play an anti-inflammatory role and can be involved in resolution of immune responses as well as processes such as wound healing [77]. The impact of macrophages on lymphangiogenesis in the mammary gland has been primarily characterized during postpartum involution. During postpartum involution, macrophages express pro-lymphangiogenic stimuli and are primarily M2-polarized [42, 79, 80]. A conditional macrophage knockout mouse model (Macrophage colony-stimulating factor 1 (CSF1) receptor knockout) initiated during involution showed a delay in postpartum involution through suppression of epithelial cell apoptosis and adipocyte repopulation [80]. However, the impact of macrophages on pubertal lymphangiogenesis in the mammary gland has yet to be identified. Estrogen, a steroid hormone mostly associated with female reproductive development, is known to regulate ductal elongation during puberty via its receptors ERα and ERβ on mammary epithelial cells. Estrogen has also been shown to recruit macrophages to the developing mammary gland, likely through AREG and EGFR signaling [81]. Additionally, estrogen promotes postpartum involution through increased mammary inflammation, cell apoptosis, and adipocyte repopulation [82]. Therefore, estrogen could regulate macrophage involvement during pubertal development of the mammary gland. Furthermore, lymphatic endothelial cells express ERα, and estrogen can promote gene expression of lymphatic specific markers, such as *Prox1* [83]. Beyond this, estrogen signaling has not been linked to lymphangiogenesis or lymphatic vascular stability, and therefore may be of interest to determine its contribution to regulation of macrophages in remodeling events such as during pubertal development and lymphangiogenesis.

Since VEGFC recruits macrophages to the mammary gland during tumor development [68], this mechanism could also be at play during pubertal development of the mammary gland. During pubertal ductal tree expansion, macrophages associate with developing TEBs for ductal elongation [84]. Consistent with a role for macrophages in ductal outgrowth, a macrophage-deficient mouse model (CSF1 knockout) showed defective outgrowth and branching during development [85]. Additionally, macrophages in the pubertal mammary gland are found to express M2-polarized macrophage marker, ARG1 [81]. Although the primary source of pro-lymphangiogenic signals in the developing ductal tree were found to be myoepithelial cells and not hematopoietic cells, which would include macrophages, macrophages could still be an important source of matrix remodeling signaling for lymphatic invasion in the mammary gland [63]. For example, bone marrow-derived macrophages (BMDMs) can express LYVE1, PROX1, and PDPN, and form lymphatic-like structures in vitro [47] and LYVE1 + PROX1 + PDPN + bone marrow derived cells undergo lymphatic vascular mimicry during neo-lymphangiogenesis in VEGFC-expressing pancreatic tumors in mice [47]. We have termed this macrophage-LEC interaction “macphatics”. Our group identified macphatics during mammary gland involution in mice as well as in breast cancer associated lymphatic vasculature in mice and in human tissues [41, 42, 62]. Other groups have shown macrophages associated with lymphatics throughout development, mainly through mouse models. For example, myeloid-derived macrophages colocalized with lymphatic vessels in the heart during embryonic development [86]; PDPN + cells derived from bone marrow with increased lymphangiogenic marker expression showed incorporation in lymphatics vessels in the cornea, wounded skin, and peritumoral melanoma tissues [87, 88]; and CD11b + macrophages formed tube-like structures and expressed lymphatic markers during inflammation-associated lymphangiogenesis in the cornea [43]. Furthermore, cultured macrophages have been polarized in vitro toward VEGFR3-expressing lymphatic endothelial cell progenitors and then integrated into lymphatic vessels in an inflammatory mouse model [44]. We hypothesize similar processes may occur during pubertal mammary gland lymphangiogenesis with a role for macrophages as a pseudo-endothelial cell in addition to promoting ductal elongation. Additionally, macrophage-vascular mimicry and macphatics formation have not been investigated in the context of lymphvasculogenesis (defined above), which occurs from the hemogenic endothelium and is an origin of macrophages during embryogenesis. Although macrophage origin and differentiation outside their association with lymphatic vasculature is beyond the scope of this review, we wanted to bring attention to the similar hemogenic origin.

It is also unknown whether macrophages that express pro-lymphangiogenic markers are polarized to M1-like or M2-like phenotypes. It is widely regarded that tumor-promotional M2-like macrophages promote lymphangiogenesis due to expression of pro-lymphangiogenic factors. However, the involvement of M1- or M2-like macrophages in vascular mimicry have yet to be investigated. Our data suggests that PoEMs, which can form macphatics, more closely resemble M2-like macrophages [42]. Meanwhile, identification of the role of differentially polarized macrophages in in vitro angiogenic assays shows that M1-like macrophages are more likely to contribute to vessel formation and sprouting while M2-like macrophages contribute toward stability [89]. However, both M1- and M2-like macrophage populations are present in in vivo vascular grafts in mice with no discernable difference in contribution between macrophage subtypes [89]. Since the primary population of macrophages during postpartum involution and pubertal development have been identified to be M2-like, how M2-like macrophages contribute to lymphangiogenesis during development in the mammary gland compared to M1-like macrophages remains to be investigated. Additionally, while TAMs have been shown to express M1-like expression factors, such as TNFα [46, 78], TAMs in the mammary gland are typically identified as M2-polarized with similar expression of pro-tumorigenic factors, such as VEGFC/D, matrix remodeling enzymes (MMPs), and ANG2 [46]. In pancreatic cancer, an increase in M2-polarized TAMs (CD163 + CD68+) correlated with an increased in LVD and poor prognosis in patients compared to unpolarized TAMs (CD69+) [90]. In a mouse model of lung adenocarcinoma, most TAMs were found to be M2-like (CD68 + CD206+) and increased M2-like TAMs correlated with increased LVD [91]. However, it has also been shown that there was no correlation with VEGFC-expressing TAMs and LVD in breast cancer patients [92]; however, these VEGFC-expressing TAMs did positively correlate with VEGFC-expressing tumors [92]. Taken together, M2-like macrophages contribute more greatly to lymphangiogenesis overall, yet this also demonstrates the need for further investigation in the mammary gland on how macrophages aid in pro-lymphangiogenic signaling versus vascular mimicry, and if this changes with macrophage polarization.

## STAT Signaling

Signal transducer and activator of transcription (STAT) molecules are cytoplasmic transcription factors that regulate gene transcription after being phosphorylated and activated via cytokine signaling through Janus kinase (Jak) receptors [93, 94]. While there are seven described mammalian STAT molecules, here we discuss three STATs that have been implicated in mammary gland morphogenesis: STAT3, STAT5, and STAT1. STAT3 expression is increased in the mouse mammary epithelium during postpartum involution, first by inducing an apoptotic response during the first phase of postpartum involution, followed by inducing an immune response and macrophage polarization during the second phase [95]. STAT3 is also implicated in VEGFC/VEGFR3 signaling during breast cancer. High expression levels of STAT3 in invasive breast cancer correlates with lymph node metastases, VEGFC/D, and VEGFR3 expression [96]; therefore, STAT3 may play an indirect role in promoting lymphangiogenesis during involution. Additionally, VEGF/VEGFR2 signaling has been shown to promote STAT3 activation leading to self-renewal of breast cancer cells in vitro [97] and VEGFA induces LEC migration and tube formation via STAT3 signaling [98].

Two additional members of the STAT family are isoforms of STAT5: STAT5A and STAT5B. Both have been implicated in mammary gland development and breast cancer progression. A *Stat5a* knockout mouse model shows reduced secondary and side branching during pubertal development in the mammary gland as well as reduced proliferation and differentiation of epithelial cells [99]. A *Stat5b* knockout mouse model also showed impaired mammary gland development, inconsistent viable pregnancies, and insufficient milk proteins during lactation [100]. Since STAT5A and STAT5B can heterodimerize as well as homodimerize as transcription factors, STAT5A and STAT5B are typically genetically manipulated together in mouse models. Dual STAT5A and 5B deletion in a mouse model prior to pregnancy showed inhibition of alveolar progenitor cell proliferation, differentiation, and cell survival during pregnancy [101]. The production of a STAT5 pro-survival signal allows for inactivation of STAT3 before involution can begin. Therefore, the opposing regulation of STAT3 and STAT5 during mammary morphogenic events could contribute to a regulation of lymphangiogenesis, which is yet to be investigated. Interestingly, in a mouse model, knocking out STAT5 expression in macrophages has been shown to increase expression of tissue remodeling factors, including collagen and VEGFA [102]. Furthermore, loss of STAT5 expression in these macrophages showed an increase in tumor size and lung metastasis [102]. Therefore, STAT expression in macrophages could also impact the role of macrophages in promoting lymphangiogenesis. Finally, STAT1 is phosphorylated and active in mature virgin glands and after involution [94], suggesting a similar signaling mechanism at play during puberty and involution, but not during pregnancy or lactation. In summary, STAT signaling between puberty and involution in mammary epithelial cells serves different purposes depending on the function of the morphological event, with pro-survival signaling versus apoptotic induction, respectively. However, there may be extrinsic mechanisms that are similar in regulation of ductal morphology that impacts corresponding lymphangiogenesis. Overall, this reflection provides support for further investigation into comparing STAT signaling in pubertal development to involution to determine how STAT signaling contributes to lymphangiogenesis during puberty and postpartum involution.

## COX2/PGE2 Signaling

Cyclooxygenase 2, or COX2, is an important regulator of the lymphatic-associated “wound-healing” components during mammary gland postpartum involution. In a mouse model of postpartum involution, inhibition of COX2 via celecoxib during postpartum involution reduced LVD without significantly interfering with mammary gland involution morphology [67]. Additionally, COX2 and its downstream metabolite, prostaglandin E2 (PGE2), have been shown to promote lymphangiogenesis in the breast tumor microenvironment [103, 104]. VEGFC and COX2 are significantly correlated with lymphangiogenesis and a poor prognosis of invasive breast cancer, including increased lymph node metastasis and worse overall survival of breast cancer patients [105]. In prostate cancer, COX2 correlates with VEGFC expression, tumor lymphangiogenesis, and lymphatic metastasis [106]. Furthermore, VEGFD promotes lymphatic vessel dilation through PGE2 [107], serving as a COX2-mediated mechanism for lymphatic tumor cell spread. Although little is known of the role of COX2 during pubertal mammary gland development, an MMTV-COX2 transgenic mouse crossed with a PGE2 receptor knockout mouse (Ep2-/-) shows normal ductal development compared to the expected mammary hyperplasia in a wild-type cross. This normal phenotype suggests that pubertal mammary ductal expansion is also regulated through PGE2 signaling via this receptor [108]. Therefore, a similar mechanism of promoting lymphangiogenesis through COX2 found during involution could be occurring during pubertal development, since increased ductal expansion during puberty correlates with increased LVD [63]. Finally, PGE2 may polarize macrophages to be M2-like [109], which are found in both the pubertal mammary gland and postpartum involuting mammary gland [79, 81]. Therefore, macrophages may be transformed to promote lymphangiogenesis through similar mechanisms mediated by PGE2.

**In summary**, we have detailed that similar pro-lymphangiogenic signaling mechanisms, which have been characterized during postpartum involution, are also at play during pubertal development (Fig. 1).We reported that the stromal remodeling events during postpartum involution, such as increased COX2 expression and collagen deposition, creates a favorable microenvironment for breast cancer progression [110], easing access for tumor cells to metastasize through neo-lymphangiogenesis and macphatic formation. We propose that the macrophage-involved lymphangiogenic events, such as macphatics formation, could also be occurring during pubertal lymphangiogenesis. Therefore, lymphangiogenic signaling during pubertal mammary gland development should be studied, as this could provide insight to similar mechanisms occurring during tumorigenesis especially for nulliparous women who develop breast cancer.

## Connecting Mammary Gland Lymphatic Development to Lymphatics in Breast Cancer

Breast cancer is the most common diagnosed cancer in women in the US and is the second leading cause of cancer-related deaths in women [111]. Breast cancer is also the primary tumor site most likely to lead to distant metastasis in women [112]. Although breast cancer metastasis can occur via blood vasculature or lymphatic vasculature, some mouse models indicate that breast cancer may preferentially metastasize through lymphatic vasculature and that passage through the lymph node may be required to enter the blood [113–115]. Additionally, stage and metastatic spread of breast cancer is initially determined with first identifying tumor cells in the draining lymph node, demonstrating the importance of studying mechanisms of lymphatic formation and structure in the mammary gland.

Angiogenesis/Lymphangiogenesis is one of the hallmarks of cancer [116]. Inducing vascular growth in tumors promotes blood supply and dilated lymphatic vessels, which is thought to allow for tumor cell migration to promote distant metastasis [117]. Tumor cell migration can also be aided by an increase in lymphatic flow from enlarged vessels. These enlarged vessels have been visualized via intravital microscopy and fluorescence photobleaching in a mouse tumor model with VEGFC overexpression [118]. VEGF expression is found to be increased in many solid tumors, including breast cancer [119] and expressed by tumor cells to promote pro-tumorigenic lymphangiogenesis [120]. As evidence that VEGFC contributes to breast cancer progression, overexpression of VEGFC in an orthotopic mouse model of breast cancer showed increased intratumoral lymphangiogenesis alongside increased metastasis to lymph node and lung [121]. Human breast cancers with a high expression of VEGFC are characterized by higher LVD in tissues biopsied from tumors, increased lymph node metastasis, distant metastasis and a worse prognosis [122]. VEGF expression can also be induced by the hypoxic environment in solid tumors via hypoxia inducible factors (HIFs) [123]. Normal developmental cues for lymphangiogenesis are also found to occur in tumor-associated lymphatic vessels, including expression of NRP2, which is typically expressed in prenatal lymphangiogenesis [124]. Similarly, vasculogenic mimicry, which we have identified during mammary gland involution, has been found in inflammatory breast cancer and ductal breast carcinoma [125]. Therefore, further characterizing mechanisms of lymphangiogenesis in normal mammary gland development may provide insight on tumor-associated lymphangiogenesis in the mammary gland and in breast cancer.

## Conclusions

**Fig. 2:**
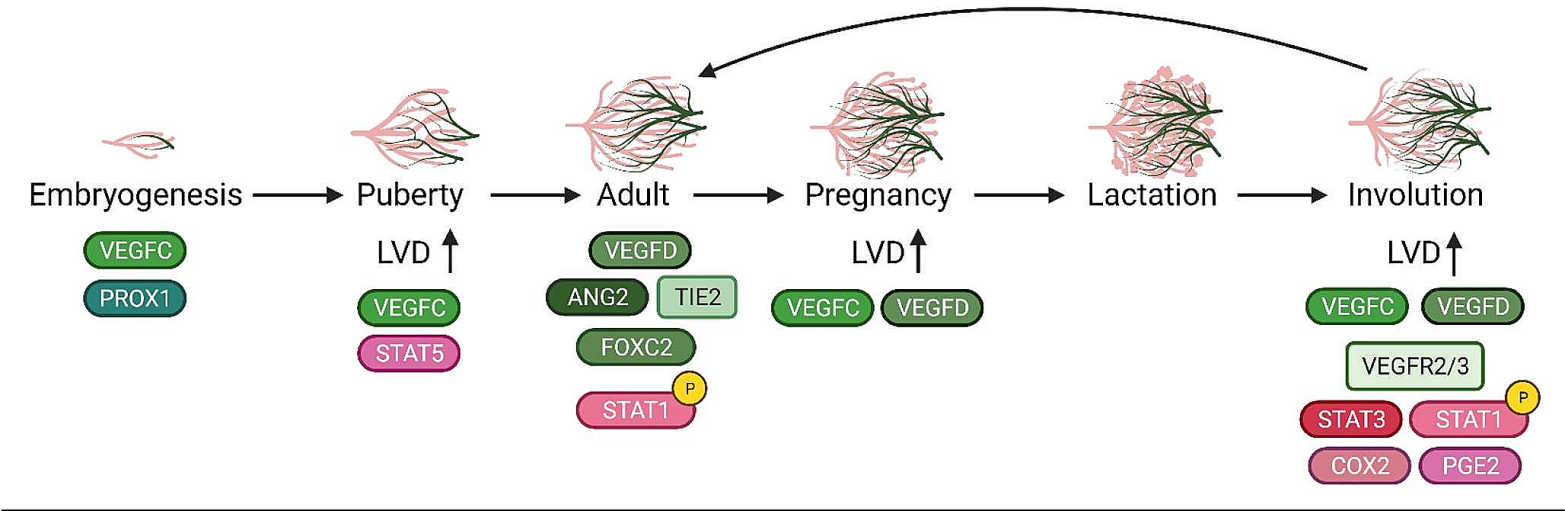
Model of lymphangiogenic signals throughout mammary gland development. As the mammary gland undergoes remodeling during major morphological events, the main lymphangiogenic factors are found changing, along with lymphatic vessel density (LVD). These factors are listed under each major event during mammary gland development and their representative ductal tree (pink) and lymphatic vascular structure (green). The colors of the factors demonstrate the cells it is acting on during each event (green for lymphatic endothelial cells, pink for mammary epithelial cells). This figure shows the signaling contributing to maintenance of lymphatic vasculature structure and lymphangiogenesis that has been previously investigated during mammary morphogenesis while also conveying the gaps in knowledge during these events, mainly during puberty, pregnancy, and lactation; meanwhile, this has been vastly characterized in the normal mammary gland (with normal lymphatic structure) and during postpartum involution. Figure was created using BioRender.com.

In this review, we described what is known about lymphatic vasculature development throughout mammary gland development and its changing morphology. The establishment of lymphatics during these functional events may also contribute to the potential of tumor cell metastasis in breast cancer progression. Figure 2 shows the currently known factors that aid in characterizing lymphatic vasculature structure and lymphangiogenesis throughout mammary gland development, while also conveying gaps in this area—mainly for puberty, pregnancy, and lactation. Meanwhile, lymphatics during postpartum involution have been shown to help reestablish a “normal” mammary gland environment but could also be highjacked by a tumor during this phase to aid in metastatic spread. Since lymphatic vasculature in the mammary gland correlates with ductal morphology, investigating lymphangiogenesis during pubertal development could further elucidate mechanisms of tumor lymphangiogenesis, especially in nulliparous women. We have also described similar signaling mechanisms in pubertal development in regard to lymphangiogenesis as during postpartum involution, demonstrating a starting point for these future studies in pubertal development. Macrophages during postpartum involution and tumorigenesis also promote lymphangiogenesis, yet a similar role for macrophages in pubertal development remains to be explored. Overall, characterizing the role and structure of the lymphatic network, and subsequent changes during mammary morphogenesis, will help us further understand mechanisms of lymphangiogenesis for tumor progression. This may lead to insights into tumor microenvironments that carry a higher risk of metastasis and factors that can then be targeted to reduce metastatic progression in breast cancer.
